# The effect of multidisciplinary rehabilitation on brain structure and cognition in Huntington's disease: an exploratory study

**DOI:** 10.1002/brb3.312

**Published:** 2015-01-15

**Authors:** Travis M Cruickshank, Jennifer A Thompson, Juan F Domínguez D, Alvaro P Reyes, Mike Bynevelt, Nellie Georgiou-Karistianis, Roger A Barker, Mel R Ziman

**Affiliations:** 1 School of Medical Sciences, Edith Cowan UniversityPerth, Western Australia, Australia; 2 School of Psychological Sciences, Monash UniversityMelbourne, Victoria, Australia; 3 Department of Surgery, UWA and Neurological Intervention and Imaging Service of Western AustraliaPerth, Western Australia, Australia; 4 John van Geest Centre for Brain RepairCambridge, U.K; 5 School of Pathology and Laboratory Medicine, University of Western AustraliaPerth, Western Australia, Australia

**Keywords:** Cognition, executive function, Huntington's disease, neuropathology, rehabilitation

## Abstract

**Background:**

There is a wealth of evidence detailing gray matter degeneration and loss of cognitive function over time in individuals with Huntington's disease (HD). Efforts to attenuate disease-related brain and cognitive changes have been unsuccessful to date. Multidisciplinary rehabilitation, comprising motor and cognitive intervention, has been shown to positively impact on functional capacity, depression, quality of life and some aspects of cognition in individuals with HD. This exploratory study aimed to evaluate, for the first time, whether multidisciplinary rehabilitation can slow further deterioration of disease-related brain changes and related cognitive deficits in individuals with manifest HD.

**Methods:**

Fifteen participants who manifest HD undertook a multidisciplinary rehabilitation intervention spanning 9 months. The intervention consisted of once-weekly supervised clinical exercise, thrice-weekly self-directed home based exercise and fortnightly occupational therapy. Participants were assessed using MR imaging and validated cognitive measures at baseline and after 9 months.

**Results:**

Participants displayed significantly increased gray matter volume in the right caudate and bilaterally in the dorsolateral prefrontal cortex after 9 months of multidisciplinary rehabilitation. Volumetric increases in gray matter were accompanied by significant improvements in verbal learning and memory (Hopkins Verbal Learning-Test). A significant association was found between gray matter volume increases in the dorsolateral prefrontal cortex and performance on verbal learning and memory.

**Conclusions:**

This study provides preliminary evidence that multidisciplinary rehabilitation positively impacts on gray matter changes and cognitive functions relating to verbal learning and memory in individuals with manifest HD. Larger controlled trials are required to confirm these preliminary findings.

## Introduction

Huntington's disease (HD) is a degenerative disorder of the nervous system caused by an unstable cytosine-adenine-guanine (CAG) expansion in exon 1 of the HTT gene (MacDonald et al. 1993). Despite progress, there is still no cure and available drug agents only provide partial relief of motor and psychiatric symptoms. There is, therefore, an urgent need to trial treatments that can impact on disease-related brain changes and clinical aspects of HD.

Over the last decade, parcellation and voxel based morphometry (VBM) imaging studies have shown evidence of grey matter (GM) degeneration in cortical and subcortical brain structures in HD (Hobbs et al. 2011; Dominguez et al. 2013; Georgiou-Karistianis et al. 2013a). Degeneration of GM is particularly pronounced in the striatum, commencing up to 20 years prior to clinical onset (Georgiou-Karistianis et al. 2013a; Tabrizi et al. 2013). Over the course of the disease, GM loss becomes more widespread, with atrophy also observed in frontal and occipital cortices (Dominguez et al. 2013; Tabrizi et al. 2013).

Deficits in cognitive function also arise in HD, even prior to diagnosis, presumably as a result of the neurodegenerative processes (Stout et al. 2012). In early HD, there are documented deficits in attention (Georgiou-Karistianis et al. 2012), psychomotor speed (Stout et al. 2012), working memory (Stout et al. 2012), planning and inhibition (Ho et al. 2003). In the absence of effective treatments, these deficits worsen over time, negatively impacting on functional independence and quality of life (Eddy and Rickards 2013).

The loss of GM has been shown to correlate with a decline in cognitive performance in HD. Scahill et al. (2013) have shown that loss of GM in cortical and subcortical structures significantly correlates with poorer performance on emotional recognition, working memory and odor identification tasks. Harrington et al. (2014) have further shown that degeneration of fronto-striatal and fronto-parietal structures correlates with poorer performance on attention, processing speed, verbal learning and memory and emotional recognition tasks.

Recent evidence suggests that lifestyle factors significantly influence disease-related brain and cognitive changes in HD. Bonner-Jackson et al. (2013) have shown that greater cognitive reserve (computed as the composite of innate intelligence and educational level) is associated with a slower rate of volume loss in the caudate nucleus and putamen and greater preservation of cognitive function in premanifest HD. Moreover, higher education status is significantly associated with a better cognitive outcome on the Unified Huntington's Disease Rating Scale (UHDRS) in manifest HD (López-Sendón et al. 2011). Finally, lifestyle passivity has been shown to significantly influence the onset of symptoms in HD (Trembath et al. 2010). Treatment strategies that enrich lifestyle may impact on disease-related brain changes and a loss of cognitive function in HD and warrant further investigation.

Previous studies have shown that environmental enrichment can preserve peristriatal structures and cognitive function in HD rodent models (van Dellen et al. 2000; Wood et al. 2010). Moreover, lifestyle interventions, such as multidisciplinary rehabilitation, have been shown to improve aspects of cognition, functional capacity, depression and quality of life (Zinzi et al. 2007; Veenhuizen et al. 2011; Piira et al. 2013; Thompson et al. 2013). When assessed separately, cognitive and motor interventions have also been reported to increase hippocampal, GM and white matter volume in the elderly and those with neurodegenerative disorders (Erickson et al. 2011; Burciu et al. 2013; Bonzano et al. 2014; Kühn et al. 2014).

The outlined findings informed our decision to evaluate the utility of multidisciplinary rehabilitation on disease-related brain changes and cognitive function in manifest HD. Specifically, we evaluated the effects of multidisciplinary rehabilitation on attenuating GM loss and associated declines in cognitive function. We hypothesized that multidisciplinary rehabilitation would increase GM volume in dorsolateral prefrontal cortex (DLPFC), striatum, and hippocampus structures that are known to be functionally relevant to cognitive function. In addition, we expected GM volume increases to be associated with better cognitive outcomes.

## Materials and Methods

### Study design

The present investigation was a 9 month exploratory study on the effects of multidisciplinary rehabilitation on brain structure and cognition in individuals with manifest HD. The duration of the intervention was chosen for two reasons: (1) structural changes can be detected in individuals with manifest HD after 6 months (Henley et al. 2006), and (2) evidence has shown that rehabilitation interventions can have favorable effects on brain structure after 2 weeks (Burciu et al. 2013).

### Study approval, registration, and patient consent

Ethical approval was granted by the Edith Cowan University and North Metropolitan Area Mental Health Service (NMAMHS) Human Research Ethics Committees. Written informed consent was provided by all participants.

### Participants

Fifteen participants with manifest HD were recruited using the North Metropolitan Area Mental Health Service Neuroscience Unit Database. Inclusion criteria included a family history of HD, a positive genetic test for the HD mutation (CAG >39), manifest disease [Unified Huntington's Disease Rating Scale-Total Motor Score (UHDRS-TMS) >5], the capacity to follow written or verbal instruction, the ability to perform submaximal aerobic and resistive exercise and aged 18 years or older. Participants were excluded if they suffered from recent drug or alcohol abuse, had a confounding neurological condition or concomitant physical, cardiovascular or respiratory condition which contraindicated exercise. Medication adjustments were recorded routinely throughout the trial (see Table 1).

**Table 1:** Participant demographics

No	Gender	CAG length	Age	Disease Duration (Years)	DBS	UHDRS-TMS	Medication (baseline)	Medication (during)
1	Male	46	57	7.6	596	45	Aripiprazole, Mirtazapine, Escitalopram	Aripiprazole Escitalopram
2	Male	42	71	9.5	461.5	59	Clonazepam, OlanzapineAmantadine, Mirtazapine	Clonazepam, OlanzapineAmantadine, Mirtazapine
3	Female	46	51	2.3	535.5	18	Venlafaxine, Mirtazapine, Olanzapine	Setraline, Creatine, CoQ10, Venlafaxine, Mirtazapine, Olanzapine
4	Male	45	47	1.8	446.5	52	Aripiprazole, Olanzapine, Mirtazapine, Escitalopram, Nitrazepam	Aripiprazole, Olanzapine, Mirtazapine, Escitalopram, Nitrazepam, Benzhexol
5	Female	46	45	4.2	472.5	36	Olanzapine, Escitalopram, CoQ10	Olanzapine, Escitalopram, CoQ10
6	Female	44	54	0.9	459	19	–	Olanzapine, Escitalopram
7	Female	41	50	0.6	275	25	Mirtazapine, Escitalopram, Aripiprazole, Lorazepam	Mirtazapine, Escitalopram, Aripiprazole, Lorazepam, Tetrabenazine, Propranolol
8	Male	44	48	1.4	408	58	Aripiprazole, Gabapentin, Escitalopram, Olanzapine	Amantadine, Clonazepam, Amantadine, Gabapentin, Pramipexole
9	Female	44	50	10.5	433.5	44	Tetrabenazine	Fluoxetine, Tetrabenazine, Actonel
10	Female	39	49	3.3	175	39	–	–
11	Male	41	61	0.9	335.5	13	–	–
12	Female	43	56	1.4	427.5	32	Haloperidol, Paroxetine	Haloperidol, Paroxetine
13	Male	41	53	17.3	297	5	CoQ10	CoQ10
14	Male	44	48	1	408	12	Escitalopram	Aripiprazole, Escitalopram
15	Male	40	68	6.7	310.5	17	Prazosin	Aripiprazole, Atenolol, Atorvastatin, Clonazepam, Clopidogrel, Quetiapine,
Summary	8M/7F	43.6 ± 2.2	52.5 ± 6.6	4.6 ± 4.8	402.7 ± 107.7	31.6 ± 17.5	NA	NA

DBS, Disease Burden Score (age × [CAG−35.5]), UHDRS-TMS, Unified Huntington's Disease Rating Scale-Total Motor Score.

### Multidisciplinary rehabilitation intervention

The intervention was designed after baseline assessment of the participants by an experienced interdisciplinary team consisting of physical therapists, exercise physiologists, occupational therapists, and strength and conditioning specialists. The intervention consisted of a clinical exercise program, a home-based exercise program and fortnightly occupational therapy. The clinical exercise program consisted of supervised weekly aerobic and resistance exercises for an hour. The home-based exercise program involved thrice weekly self-directed muscle strengthening and fine motor exercises for an hour. Occupational therapy consisted of a variety of paper and pencil, verbal planning, memory, and problem solving exercises designed to enhance cognition and executive function (see the online Appendix S1 for specific details on the multidisciplinary rehabilitation intervention). Adherence to clinical exercise and occupational therapy sessions were recorded by clinical exercise specialists and occupational therapists using a training diary. Adherence to the home-based exercise sessions were recorded by patients using a provided training diary.

### Outcome measures

#### Magnetic resonance imaging

Structural MR (magnetic resonance) images from 15 participants were acquired at baseline and 9-month follow-up using a 3T Philips Achieva Scanner and a Philips 8 – channel head coil (Philips Healthcare. Best, The Netherlands). Structural scans consisted of a T_1_ 3D Turbo Field Echo (TFE) scan (400 × 400, 130 slices, 1 × 1 × 1 mm voxels, TR = 5.8 msec, TE = 2.7 msec).

Voxel-based morphometry (VBM) was performed on structural MR images to determine increases and decreases in GM volume between baseline and 9 months. As implemented in FSL-VBM Version 1.1, the VBM (Douaud et al. 2007), protocol included removal of nonbrain tissue from each participant's images, tissue segmentation into GM, spatial normalization (nonlinearly to MNI 152) at 2 × 2 × 2 mm^3^ resolution and (nonlinear) registration to a right-left symmetric, study-specific GM template (average of all individual grey matter images). These images were modulated and then smoothed with a Gaussian kernel of ∽4.6 mm full width half maximum (FWHM).

#### Cognitive and executive function measures

Cognitive performance was evaluated at baseline and at 9 months using a variety of cognitive measures previously shown to be sensitive in HD (Stout et al. 2012; Tabrizi et al. 2013). The Color Word Interference Test (CWIT) and Trail Making Test components of the Delis-Kaplan Executive Function System (D-KEFS) (Delis et al. 2001, 2004) were used to examine response inhibition and cognitive flexibility. The Symbol Digit Modalities Test (SDMT) (Smith 1982) was used to examine information processing speed and attention. Verbal learning and memory were examined using the Hopkins Verbal Learning Test-Revised (HVLT-R)(Brandt 1991). All cognitive assessments were performed by cognitive raters blinded to the treatment condition.

### Statistics

Demographic data are given as means and standard deviations. We used linear regression to estimate the increase or decrease in GM volume between baseline and 9 months. The regression model included separate explanatory variables for each participant (for each subject's mean effect) and age. Analysis was focused on regions-of-interest (ROIs) defined a priori based on previous studies in HD shown to be functionally relevant in terms of cognitive capacity (as reflected in episodic memory performance). ROIs included the striatum, hippocampus, and dorsolateral prefrontal cortex (DLPFC). Inferential statistics were carried out using a nonparametric permutation method (as implemented by FSL's randomise tool). Only clusters with >10 contiguous voxels at a significance level of *P < *0.05 were considered to be indicative of significant longitudinal change. As we adopted an exploratory analysis strategy with ROIs clearly defined a priori, no correction for multiple comparisons was applied. GM volume change was also evaluated beyond the ROIs. In this case, maps were thresholded at *P < *0.01 (uncorrected) and voxels were considered significant within clusters of >10 contiguous voxels. The normality of cognitive data was assessed using the Schapiro-Wilk test. Changes in cognitive performance were assessed using mean values at baseline and at 9 months with paired *t*-tests. Statistical significance was set at (*P *≤* *0.05). All statistical analyses were performed using STATA 9.1 (Stata Corp, 4905 Lakeway Dr, TX). We then investigated the functional relevance of change in GM volume in the ROIs, as reflected by associations between significant volume changes and significant change in performance measures from the HVLT-R (follow-up score minus baseline score): total recall, delayed recall, retention, and the recognition discrimination index (RDI). The HVLT-R was chosen as dysfunction in recall and recognition memory is an important clinical feature of HD (Montoya et al. 2006). In order to quantify GM volume change, we created a single difference image for each participant by subtracting the follow-up from the baseline smoothed, modulated image generated by the VBM protocol. The relationship between volume change in ROIs and change in cognitive function was then assessed voxel-wise by means of FSL's randomize tool. Age was included as a covariate in all analyses.

## Results

### Demographics

Table 1 displays demographic data and information on disease duration, disease burden and severity of motor abnormalities. Participants displayed high adherence to the supervised clinical program (84.2%), moderate adherence to the home-based program (58.6%) and high adherence to occupational therapy sessions (79.2%).

### Structural brain changes

**Figure 1:**
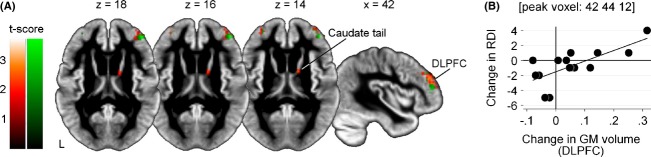
Significant GM volume changes after multidisciplinary rehabilitation in individuals with manifest HD (A) Significantly increased GM volume in the DLPFC and right caudate nucleus tail after 9 months of multidisciplinary rehabilitation in individuals with HD (red-yellow), and a significant correlation between increased GM volume in DLPFC and preserved performance on the RDI task (green). Results are displayed on the study-specific template normalized to MNI space (*P < *0.05, uncorrected). (B) Scatterplot illustrating the correlation between increased DLPFC volume at the peak voxel and preserved performance on the RDI task.

**Figure 2:**
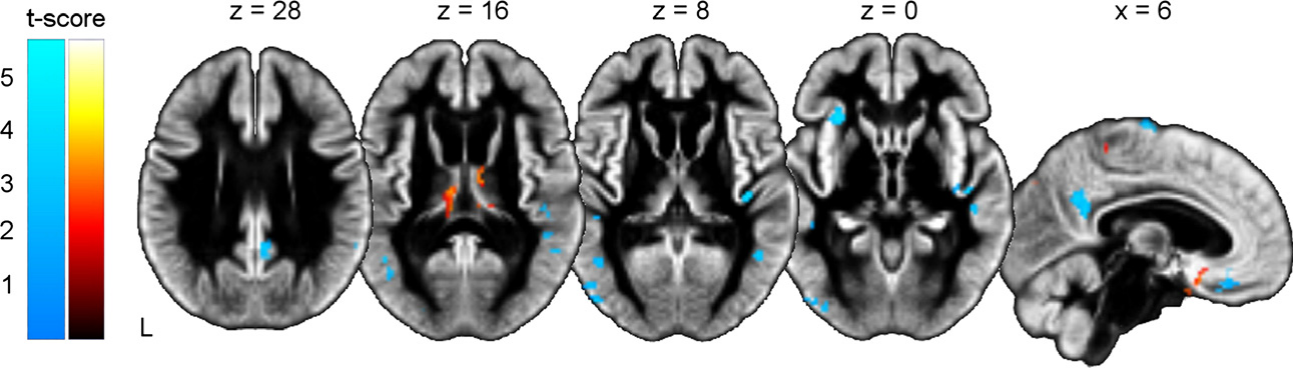
Whole brain GM volume changes in individuals with manifest HD Results of the VBM analysis beyond the ROIs after 9 months of multidisciplinary rehabilitation. GM volume loss in blue; GM volume increases in red-yellow. Results are displayed on the study-specific template normalized to MNI space (*P < *0.01, uncorrected).

Figure1 shows significant volumetric increases in GM in the DLPFC bilaterally and in the tail of the right caudate nucleus after multidisciplinary rehabilitation. All remaining ROIs, including the right hippocampus, left putamen, and accumbens showed GM volume loss. Beyond these ROIs, changes in GM volume were also observed. The superior thalami, left inferior temporal pole, right subcallosal cortex, and parasagittal primary motor areas exhibited increases in GM volume. By contrast, the left anterior insula, right posterior cingulate/precuneus, left lateral occipital cortex, subcallosal cortex, and focal areas in the temporal cortex bilaterally showed GM volume loss (Fig.2), consistent with previous neuroimaging studies in individuals with HD (Tabrizi et al. 2011, 2012, 2013; Dominguez et al. 2013; Georgiou-Karistianis et al. 2013a).

### Cognitive and executive function changes

Significant improvement was observed on the delayed recall (number of words recalled after delay) component of the HVLT-R after 9 months of multidisciplinary rehabilitation (see Table 2). No significant changes were found for CWIT, TMT, and SDMT outcomes after 9 months of multidisciplinary rehabilitation (see Table 2).

**Table 2:** Changes in cognitive function after 9 months of multidisciplinary rehabilitation in individuals with manifest HD

Outcome measures	Baseline (*n* = 15)	Post-trial (*n* = 15)	*P* value
CWIT			
Color naming	48.35 ± 18.86	52.35 ± 22.57	0.0999
Word reading	34.00 ± 10.97	35.35 ± 9.77	0.3249
Inhibition	91.00 ± 39.25	93.57 ± 41.39	0.4525
TMT			
Visual scanning	38.76 ± 15.76	43.52 ± 15.98	0.1149
Number sequencing	55.73 ± 19.87	61.80 ± 23.49	0.0507
Letter sequencing	61.92 ± 34.05	66.21 ± 30.77	0.1262
Motor speed	58.75 ± 27.39	62.31 ± 25.90	0.2433
HVLT-R			
Free recall	17.66 ± 5.56	16.73 ± 6.21	0.2019
Delayed recall	4.92 ± 2.36	6.28 ± 3.14	0.0130*
Retention	76.16 ± 29.21	81.22 ± 27.32	0.1866
Recognition	8.06 ± 3.08	8.93 ± 2.34	0.0793
SDMT			
Correct written	27.00 ± 10.25	26.78 ± 9.96	0.4525
Correct oral	31.00 ± 14.17	28.46 ± 15.59	0.1374

CWIT, Color Word Interference Test; TMT, Trail Making Trials; HVLT-R, Hopkins Verbal Learning Test-Revised; SDMT, Symbol Digits Modalities Test. Significance was set at ^*^P < 0.05.

### Correlation analyses

Increased GM volume in the DLPFC (bilaterally) was found to be significantly associated with preserved performance on the RDI of the HVLT-R (see Fig.1).

## Discussion

This exploratory investigation has shown that multidisciplinary rehabilitation is capable of increasing GM volume and enhancing some aspects of cognitive function in HD. Specifically, we found evidence of increased GM volume in the right caudate and bilaterally in the DLPFC, as well as an improvement in verbal learning and memory after 9 months of multidisciplinary rehabilitation. We also found a significant association between increased GM volume in the DLPFC and preserved performance in verbal learning and memory.

Similar to previous investigations in HD, we observed GM volume loss in most cortical and subcortical brain regions (Kassubek et al. 2005; Kipps et al. 2005; Peinemann et al. 2005; Mühlau et al. 2007, 2009; Hobbs et al. 2011; Tabrizi et al. 2011, 2012, 2013; Dominguez et al. 2013; Georgiou-Karistianis et al. 2013a). In this study, however, after multidisciplinary rehabilitation, we also observed increased GM volume in the DLPFC and in the right caudate nucleus in individuals with manifest HD. While this is the first study to report such a finding, recent work has shown that cognitive reserve (computed as the composite of intelligence and educational status) influences the rate of volume loss in caudate and putamen structures in individuals with premanifest HD (Bonner-Jackson et al. 2013). Moreover, environmental enrichment has been shown to preserve peristriatal cerebral volume in the R6/1 HD mouse model (van Dellen et al. 2000). Motor and cognitive interventions have additionally been shown to increase hippocampal volume, white matter and gray matter volume as well as cortical thickness in the left middle frontal gyrus, inferior frontal gyrus, superior temporal gyrus in the elderly and those with other neurodegenerative disorders (Boyke et al. 2008; Engvig et al. 2010, 2012; Erickson et al. 2010, 2011; Lövdén et al. 2012; Burciu et al. 2013; Bonzano et al. 2014; Prosperini et al. 2014; Sehm et al. 2014). These findings provide evidence to suggest that lifestyle factors play an important role in modulating the pathology and clinical profile of HD.

The structural brain changes observed in the present study and others may reflect an increase in neurogenesis and/or favorable changes to neuronal morphology (Lazic et al. 2006; Nithianantharajah et al. 2009; Nithianantharajah and Hannan 2013). This supposition stems from compelling evidence showing that environmental enrichment can increase markers of neurogenesis within the hippocampus (Lazic et al. 2006) as well as increase the diameter of dendritic spines in the R6/1 HD mouse model (Nithianantharajah et al. 2009). Molecular and cellular mechanisms that may have encouraged the surmised neurogenesis and/or alterations in neuronal morphology in response to multidisciplinary rehabilitation include an increased expression of neurotrophins like brain-derived neurotrophic factor (BDNF), enhanced cerebral angiogenesis, and a decrease in elevated circulating glucocorticoids (i.e. cortisol) (Rothman and Mattson 2013). BDNF enhances neurite outgrowth, synaptogenesis and cell survival, encouraging neurogenesis and experience-dependent synaptic plasticity (Rothman and Mattson 2013). Recent preclinical data suggests that BDNF-dependent neurogenesis is tightly coupled with cerebral angiogenesis (Chen et al. 2013), and that both are dynamically modulated by changes in circulating glucocorticoid levels (Weinstein et al. 2010; Shikatani et al. 2012; Gray et al. 2013). In particular, elevated glucocorticoid levels dampen cerebral angiogenesis and BDNF expression in healthy rodent's facilitating a decrease in neurogenesis (Shikatani et al. 2012; Gray et al. 2013; Rothman and Mattson 2013). It is possible that multidisciplinary rehabilitation facilitates an adaptive stress response that decreases circulating glucocorticoids, thereby enhancing cerebral angiogenesis and BDNF expression, encouraging neurogenesis and structural brain changes in HD patients.

There are currently no therapies that arrest or attenuate the progressive loss of cognitive function seen in individuals with HD. Here, we found evidence of an improvement in verbal learning and memory after 9 months of multidisciplinary rehabilitation. These findings extend on our previous work, where task-specific improvements in processing speed measures were found after a 9 month controlled investigation of multidisciplinary rehabilitation in individuals with manifest HD (Thompson et al. 2013). Moreover, these findings support experimental studies documenting improvements in cognitive performance in rodent models of HD after environmental enrichment (Wood et al. 2010, 2011). While evidence is limited in HD, an increasing number of studies are showing that motor and cognitive interventions positively impact on cognitive function in the elderly (Liu-Ambrose et al. 2010; Erickson et al. 2011; Bherer et al. 2013) and those suffering with MCI (Hampstead et al. 2011, 2012; Smith et al. 2013), MS (Solari et al. 2004; Flavia et al. 2010; Mattioli et al. 2010; Shatil et al. 2010) and PD (Sammer et al. 2006; Calleo et al. 2011; París et al. 2011). It is likely that the improvements in verbal learning and memory observed in this study resulted from the positive impact of multidisciplinary rehabilitation on caudate and DLPFC structures.

It is well-known that degeneration of GM contributes to the development of cognitive deficits and progressive loss of cognitive function (Scahill et al. 2013; Harrington et al. 2014). In this study, we found a significant association between increases in GM volume in the DLPFC and preserved performance in verbal learning and memory. This finding is not unexpected given that memory retrieval and recognition is driven primarily by DLPFC connectivity in healthy individuals and in those with HD (Georgiou-Karistianis et al. 2013b).

A number of limitations must be taken into account when considering our findings. First, there was no control group, which limits our ability to derive definitive conclusions on the efficacy of multidisciplinary rehabilitation on disease pathology and clinical features in HD. Second, the small sample of HD participants in this study makes generalizability difficult. Lastly, participants remained on medication throughout the study, which may have influenced the therapeutic response to multidisciplinary rehabilitation.

Despite these limitations, our findings provide the very first evidence that multidisciplinary rehabilitation is effective in increasing regional GM volume in cortical and subcortical brain regions in HD. Results also show that multidisciplinary rehabilitation is capable of improving some aspects of cognition over a 9-month period. Moreover, we found that increased GM volume in the DLPFC was associated with preservation of verbal learning and memory. These findings collectively indicate that neuroplasticity may still be present in HD and amenable to multidisciplinary rehabilitation. Future randomized controlled trials with larger sample sizes, longer duration interventions, more comprehensive imaging and cognitive outcomes and appropriate detraining periods are nevertheless required to confirm and expand on our preliminary findings.

## Supporting Information

**Table S1.**Exercises used in the clinical exercise program throughout the study. **Table S2.** Exercises used in the home-based exercise program throughout the study. **Table S3.** Exercises used in occupational therapy sessions throughout the study.
