# Supplementary material for: The effect of multidisciplinary rehabilitation on brain structure and cognition in Huntington's disease: an exploratory study
Source: Brain Behav. 2015 Jan 15;5(2):e00312. doi: 10.1002/brb3.312 (PMC4309878; doi:10.1002/brb3.312)
Supplement: Supplementary file 1 [file brb30005-e00312-sd1.docx]

**Supplementary Appendix**

**The Effect of Multidisciplinary Rehabilitation on Brain Structure and Cognition in Huntington’s disease: an Exploratory Study**

**Brain and Behaviour**

Travis M. Cruickshank^a*^, Jennifer A. Thompson^a*^, Juan F. Domínguez D^b^, Alvaro P. Reyes^a^, Mike Bynevelt^c^, Nellie Georgiou-Karistianis^b^, Roger A. Barker^d^ & Mel R. Ziman^a,e^

^a^ School of Medical Sciences, Edith Cowan University, Perth, Australia.

^b^ School of Psychological Sciences, Monash University, Melbourne, Australia.

^c^ Department of Surgery, UWA and Neurological Intervention and Imaging Service of Western Australia.

^d^ John van Geest Centre for Brain Repair, Cambridge, UK.

^e^ School of Pathology and Laboratory Medicine, University of Western Australia, Perth, Australia

*Authors contributed equally to the manuscript

Corresponding author: Travis Cruickshank, School of Medical Sciences, Edith Cowan University, 270 Joondalup Drive, Joondalup, Australia. tel: (+61 8) 6304 3416 fax: (+61 8) 6304 2626 email: [t.cruickshank@ecu.edu.au](mailto:t.cruickshank@ecu.edu.au)

**Supplementary Table S-1.** Exercises used in the clinical exercise program throughout the study.

| **Exercise Mode** | **Exercises Utilised** | **Multi/Single Joint Exercises** | **Duration** | **Intensity** | **Progression** |
| --- | --- | --- | --- | --- | --- |
| **Active Warm Up** | Walking (Treadmill)  Cycling (Ergometer)  Step ups | Not Applicable | 3-5 minutes | 40-60% | Increase warm up intensity  Decrease recovery period |
| **Aerobic Exercise** | Walking (Treadmill)  Cycling (Ergometer) | Not Applicable | 8-10 minutes | 60-80% | Increase intensity % (cadence/ resistance) |
| **Resistance Exercise** | Leg Press | Multi-joint | 40 minutes | 60-80%  2-4 Sets  8-12 Reps | Increase training volume (kg) |
|  | Knee Extension | Single-joint |  | 60-80%  2-4 Sets  8-12 Reps | Increase training volume (kg) |
|  | Knee Flexion | Single-joint |  | 60-80%  2-4 Sets  8-12 Reps | Increase training volume (kg) |
|  | Leg Abduction/ Adduction | Single-Joint |  | 60-80%  2-4 Sets  8-12 Reps | Increase training volume (kg) |
|  | Lat Pull Down | Multi-joint |  | 60-80%  2-4 Sets  8-12 Reps | Increase training volume (kg) |
|  | Supported Row | Multi-joint |  | 60-80%  2-4 Sets  8-12 Reps | Increase training volume (kg) |
|  | Chest Press | Multi-joint |  | 60-80%  2-4 Sets  8-12 Reps | Increase training volume (kg) |
|  | Abdominal Crunches | Single-Joint |  | 60-80%  2-4 Sets  8-12 Reps | Increase training volume (additional weight) |
| **Cool Down** | Walking (Treadmill)  Cycling (Ergometer) | Not Applicable | 3-5 minutes | 40-60% | Not Applicable |

Reps, Repetitions, kg, kilograms

**Supplementary Table S-2.** Exercises used in the home-based exercise program throughout the study.

| **Exercise Mode** | **Exercises Utilised** | **Multi/Single Joint Exercise** | **Duration** | **Progression** |
| --- | --- | --- | --- | --- |
| **Fine Motor** | Laser tracing  Button tying  Speed/Accuracy trade-off | Not Applicable | 15 minutes | Increase the difficulty of objects traced  Increase the number and vary the size of the buttons tied  Decrease shape size in the speed/accuracy trade-off tasks |
| **Resistance Exercise** | Knee Extension/Flexion | Single Joint | 45 minutes | Increase resistance (Sanctbands) |
|  | Wall Push | Multi-joint |  | Progress to push-ups on knees and then to full ROM push-ups |
|  | Leg Abduction/ Adduction | Single-joint |  | Increase resistance (Sanctbands) |
|  | Row | Multi-joint |  | Increase resistance (Sanctbands) |
|  | Abdominal Crunches | Single-joint |  | Increase time in eccentric and concentric contraction phases |

ROM, range of motion

**Supplementary Table S-3.** Exercises used in occupational therapy sessions throughout the study.

| **Exercise Modality** | **Tasks Utilised** | **Progression** |
| --- | --- | --- |
| **Daily Activities** | Cooking  Laundry  Gardening  Eating | Increase difficulty of cooking  Perform laundry without cues  Increased gardening to an independent state  Improve the use and manipulation of eating utensils |
| **Planning/Organisation** | Utilisation of a diary (written or electronic)  Planning social activities | Increase the number and difficulty of tasks throughout the day |
| **Memory** | Facial Recognition | Increase the number of faces to be recognised |
| **Problem Solving** | Sudoku  Board Puzzles  Boggle  Mastermind | Increase the difficulty of the Sudoku game  Increased the difficulty and size of puzzle  Include time constraints  Include time constraints |
